# Real-World Data on Clinical Outcomes and Treatment Management of Advanced Melanoma Patients: Single-Center Study of a Tertiary Cancer Center in Switzerland

**DOI:** 10.3390/cancers16050854

**Published:** 2024-02-20

**Authors:** Ramon Staeger, Julia M. Martínez-Gómez, Patrick Turko, Egle Ramelyte, Lukas Kraehenbuehl, Valerio Del Prete, Omar Hasan Ali, Mitchell P. Levesque, Reinhard Dummer, Mirjam C. Nägeli, Joanna Mangana

**Affiliations:** 1 Faculty of Medicine, University of Zurich, 8006 Zurich, Switzerland; 2 Department of Dermatology, University Hospital Zurich, 8091 Zurich, Switzerland

**Keywords:** melanoma, adjuvant treatment, unresectable melanoma, immune checkpoint inhibitors, targeted therapy, BRAF and MEK inhibitors, real-world evidence

## Abstract

**Simple Summary:**

In this retrospective study conducted at a tertiary cancer center, real-world outcomes from advanced melanoma patients were analyzed approximately a decade after the introduction of immune checkpoint inhibitors and BRAF/MEK-inhibitors into clinical use. The study included patients in the resectable/adjuvant (n = 331) and unresectable/metastatic setting (n = 375). Adjuvant anti-PD1 or BRAF/MEK inhibitors demonstrated 3-year relapse-free survival rates of 53% and 67.6%, respectively. Toxicity led to treatment cessation in 10.9% of cases, with no impact on relapse-free or overall survival rates unless treatment duration was <3 months. First-line treatment in the unresectable setting showed 5-year overall survival rates of 46.5% for anti-PD1, 52.4% for anti-CTLA4/PD1, and 49.2% for BRAF/MEK inhibitors. Brain metastases and elevated LDH levels substantially affected overall survival. For patients with progressive disease, the median overall survival rate dropped below two years. The study highlights real-world clinical management and outcomes of advanced melanoma patients, emphasizing the efficacy of approved treatments while addressing ongoing challenges.

**Abstract:**

Background: Immune checkpoint inhibitors (ICIs) and BRAF/MEK inhibitors (BRAF/MEKi) have drastically changed the outcomes of advanced melanoma patients in both the resectable/adjuvant and unresectable/metastatic setting. In this follow-up analysis of real-world data, we aimed to investigate the clinical management and outcomes of advanced melanoma patients in a tertiary referral center in Switzerland approximately a decade after the introduction of ICIs and BRAF/MEKi into clinical use. Moreover, we aimed to compare the results with seminal phase 3 trials and to identify areas of high unmet clinical need. Methods: This single-center retrospective cohort study analyzed the melanoma registry of the University Hospital Zurich, a tertiary cancer center in Switzerland, and included patients treated in the resectable/adjuvant (n = 331) or unresectable/metastatic setting (n = 375). Results: In the resectable setting, adjuvant anti-PD1 or BRAF/MEKi showed a 3-year relapse-free survival (RFS) of 53% and 67.6%, respectively, and the overall median RFS was 50 months. Patients with lymph node plus in-transit metastases or with distant metastases prior to commencing adjuvant treatment had a significantly reduced overall survival (OS). In 10.9% of patients, the treatment was stopped due to toxicity, which did not affect RFS/OS, unless the duration of the treatment was <3 months. Following a relapse of the disease during the first adjuvant treatment, the median progression-free survival (PFS2) was only 6.6 months; outcomes were particularly poor for relapses that were unresectable (median PFS2 3.9 months) or occurred within the first 2 months (median PFS2 2.7 months). A second adjuvant treatment for patients with resectable relapses still showed efficacy (median RFS2 43.7 months). Elevated LDH levels in patients with an unresectable relapse was correlated with a strong reduction in OS2 (HR 9.84, *p* = 0.018). In the unresectable setting, first-line anti-PD1, anti-CTLA4/PD1 combination, or BRAF/MEKi showed a 5-year OS of 46.5%, 52.4%, and 49.2%, respectively. In a multivariate analysis, elevated LDH levels or the presence of brain metastases substantially shortened OS (HR > 1.78, *p* < 0.035). There was a non-significant trend for the improved survival of patients treated with anti-CTLA4/PD1 compared to anti-PD1 (HR 0.64, *p* = 0.15). After a progression on first-line therapy, the median OS2 was reduced to below two years. Elevated LDH (HR 4.65, *p* < 0.001) levels and widespread disease with at least three metastatic sites, particularly bone metastases (HR 2.62, *p* = 0.026), affected OS2. Conclusion: Our study offers real-world insights into the clinical management, treatment patterns, and outcomes of advanced melanoma patients in both the adjuvant and unresectable setting. Early relapses in patients undergoing adjuvant treatment pose a particular challenge but these patients are generally excluded from first-line trials. The approved first-line metastatic treatments are highly effective in the real-world setting with 5-year OS rates around 50%. However, outcomes remain poor for patients with brain metastases or who fail first-line treatment.

## 1. Introduction

Immune checkpoint inhibitors (ICIs) and targeted therapy with combined BRAF and MEK inhibitors (BRAF/MEKi) have completely transformed the therapeutic landscape for patients with advanced melanoma [1,2,3,4,5,6,7,8,9]. Immunotherapy with the humanized monoclonal antibodies nivolumab and pembrolizumab, which bind to the programmed cell death protein 1 (PD1) receptor, have become the new standard of care, achieving long durable responses in about 35–40% of metastatic melanoma patients, regardless of the BRAF mutation status [3,7,9]. Additionally, the combination of anti-cytotoxic T-lymphocyte-associated protein 4 (CTLA4) or anti-lymphocyte activation gene 3 (LAG3) with anti-PD1 antibodies has further increased tumor response rates through unleashing potent immune effector mechanisms [3,8].

Similar to the unresectable setting, there has been a tremendous shift in the treatment paradigm of locoregional/resectable melanoma, where adjuvant treatment with anti-PD1 or BRAF/MEKi for the duration of one year is commonly recommended in completely resected stages 2b to 4, subject to local registration status [10,11,12,13,14,15].

The optimal treatment sequence of combined anti-CTLA4 and anti-PD1 (anti-CTLA4/PD1) inhibitors and BRAF/MEKi in patients with BRAF mutant advanced melanoma was recently investigated in the prospective randomized clinical trials DREAMseq and SECOMBIT; the latter included an additional arm investigating the planned switch to anti-CTLA4/PD1 inhibitors after an 8-week short course of BRAF/MEKi [16,17,18]. Both trials established first-line (1L) anti-CTLA4/PD1 inhibitors followed by second-line (2L) BRAF/MEKi as the preferred treatment approach with significantly improved survival rates compared to patients treated with the reverse sequence.

In our prior multicenter, real-world cohort study, we showed significantly increased overall survival (OS) rates and a reduction in hospitalization rates in patients receiving ICIs or targeted therapy (TT) agents compared to chemotherapy and identified high LDH levels as a negative prognostic factor for OS in TT-treated patients [19]. In this follow-up study, we aimed to investigate the clinical management and outcomes of patients with advanced melanoma in the metastatic/unresectable and the adjuvant setting, approximately a decade after the introduction of ICIs and BRAF/MEKi into clinical use. Secondarily, we aimed to analyze how well the phase 3 studies, which included highly selected patients due to narrow eligibility criteria, reflect real-world patient care in a tertiary referral center in Switzerland.

## 2. Materials and Methods

In this single-center retrospective cohort study, the melanoma registry of the University Hospital Zurich, Switzerland was queried. At time of data lock (18 April 2023), the registry included information on 1568 treatments from 750 individual patients with detailed demographic, clinical, and pathological annotation. Tumor stage is recorded according to the American Joint Committee on Cancer 8th edition.

**Figure 1:**
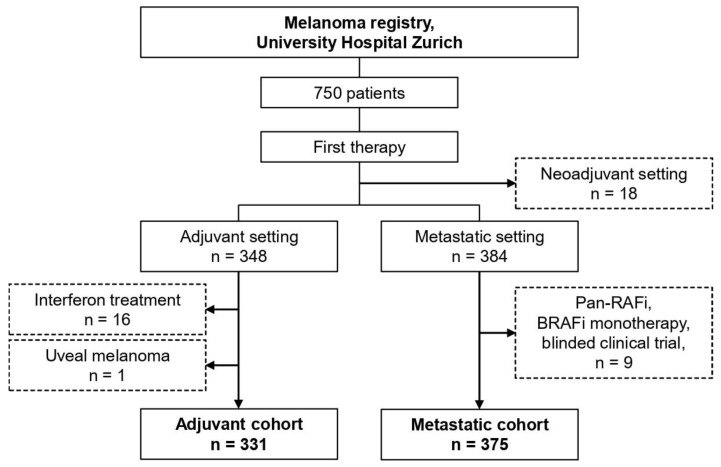
Patient selection.

We queried the frontline therapy for all 750 patients and separated into adjuvant (n = 348), metastatic/unresectable (n = 384), and neoadjuvant intention (n = 18) (Figure 1). After exclusion of interferon-based regimens (n = 16) and uveal melanoma (n = 1), the adjuvant cohort included 331 patients (Figure 1). After exclusion of Pan-RAF-inhibitor, BRAF-inhibitor monotherapy, and blinded clinical trials (n = 9), the metastatic cohort included 375 patients (Figure 1).

Alluvial plots were generated using the ggalluvial package (v0.12.5) and *ggplot2* (v3.4.4) [20,21]. For survival analysis, the R package survival (v3.5-7) was used [22]. Kaplan–Meier curves and forest plots were constructed with the survminer R package (v0.4.9) and ggplot2 (v3.4.4) [21,23]. Relapse-free survival (RFS) was calculated from the date of adjuvant therapy initiation until documented tumor relapse or death from any cause. For patients that received a second adjuvant therapy, RFS2 was calculated from the date of relapse during the first adjuvant therapy until the next documented tumor relapse or death from any cause. Progression-free survival (PFS) was calculated from the date of therapy initiation until documented tumor progression or death from any cause. PFS2 was calculated from the date of first tumor progression/relapse until the next documented tumor progression/relapse or death from any cause. Overall survival (OS) was calculated from the date of first-line (1L) therapy initiation until death from any cause. OS2 was calculated from the date of first tumor progression/relapse until death from any cause. Survival times were censored at the date of last follow-up. The log-rank test was used to compare survival times between groups and p-values were adjusted for multiple comparisons by the Benjamini–Hochberg procedure. A Cox regression model was fit to estimate hazard ratios (HR) in the univariate or multivariate analyses.

## 3. Results

### 3.1. Resectable Setting: Adjuvant Treatment

A total of 331 patients received an adjuvant treatment, of which 90.9% had a cutaneous melanoma, 7.3% had a melanoma of unknown primary (MUP), and 1.8% had a mucosal melanoma (Table 1). The majority of patients were stage 3b/c/d (79.7%), but there were a few cases of stage 2b–3a (12.4%) or resectable stage 4 (6.9%). One case was stage 2a (patient with Xeroderma pigmentosum) and two cases were stage 1b (mucosal melanoma). Lymph nodes were the most common metastatic sites (63.4% of patients), followed by in-transit (19.3%) or distant metastases (6.3%); 5.7% had a combination of lymph node plus in-transit metastases. The adjuvant treatment was most commonly anti-PD1 (74.9%), followed by enrollment in a clinical trial (8.5%), BRAF/MEKi (7.3%), anti-CTLA4 monotherapy (6.9%) or anti-CTLA4/PD1 combination (2.4%). The treatment was given for the intended timespan in 45.6% of patients or stopped earlier due to progression or toxicity in 23.9% and 10.9%, respectively.

**Figure 2:**
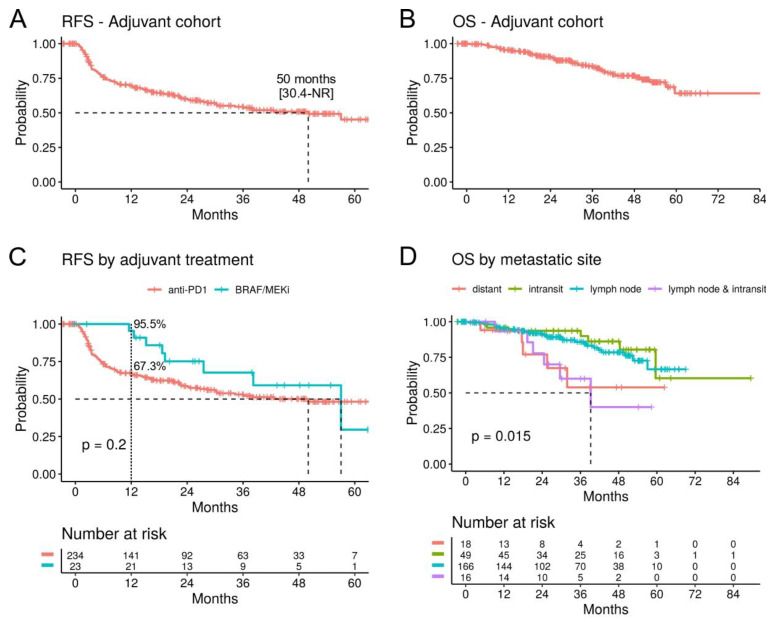
Survival analysis in the adjuvant cohort. (**A**) Kaplan–Meier curves for relapse-free survival (RFS) and (**B**) overall survival (OS) for patients with a cutaneous or unknown primary melanoma treated with anti-PD1 or BRAF/MEKi in the adjuvant setting (n = 257). (**C**) RFS stratified by adjuvant treatment. The 1-year RFS rates are indicated. (**D**) OS by site of the metastasis. (95% confidence intervals are indicated in square brackets. NR: not reached.).

For the survival analyses, we included patients with a cutaneous melanoma or MUP, treated with the approved anti-PD1 and BRAF/MEKi drugs (n = 257). The median relapse-free survival (mRFS) overall was 50 months (95% CI; 30.4-not reached (NR)) and the median overall survival (mOS) was NR (Figure 2A,B). Adjuvant treatment with anti-PD1 (n = 234) versus BRAF/MEKi (n = 23) showed no significant difference on RFS/OS (Figure 2C), but the 1-year RFS rates were 67.3% (95% CI; 61.4–73.7) vs. 95.5% (95% CI; 87.1–100) (Table 2), indicating that relapses tended to occur earlier with anti-PD1 inhibitors, while for BRAF/MEKi, the majority occurred after treatment completion.

The site of the metastasis prior to the adjuvant treatment was correlated with survival. Patients that presented with lymph node plus in-transit metastases (n = 16) had a significantly worse OS (mOS 39.2 months) compared to patients with only in-transit (n = 49) or only lymph node metastases (n = 166) (Figure 2D). The impact of lymph node plus in-transit metastases on OS (HR 3.79, *p* = 0.005) were comparable to that of distant metastases (HR 4.05, *p* = 0.009) in a multivariate analysis controlling for age, gender, treatment, and primary tumor characteristics (Supplementary Figure S1).

**Figure 3:**
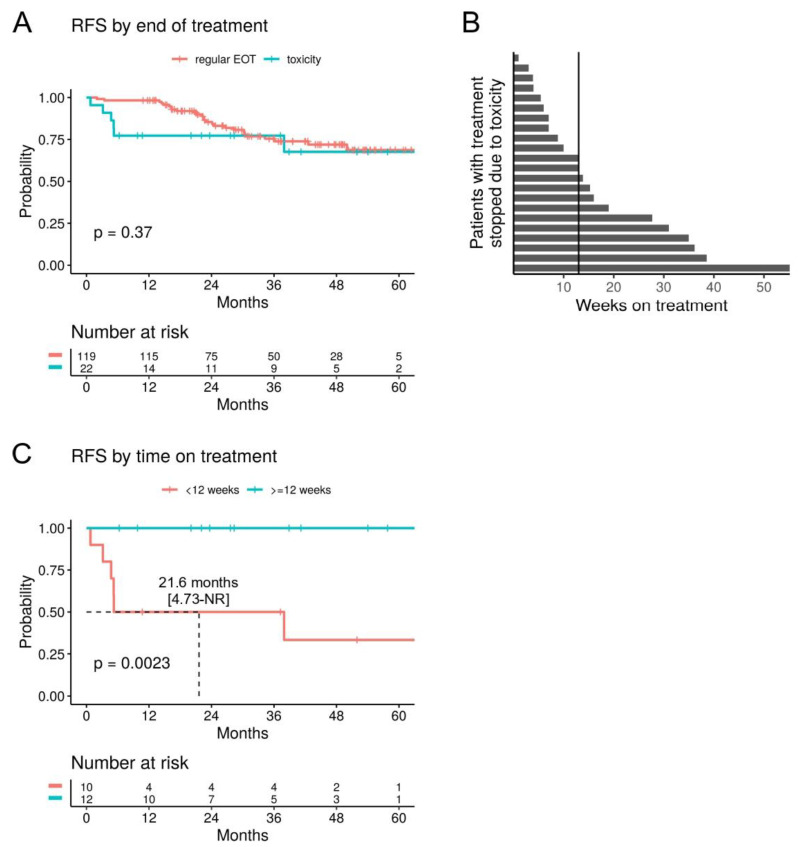
Stopping adjuvant anti-PD1 due to toxicity. (**A**) RFS of patients with adjuvant anti-PD1 that was stopped due to toxicity compared to those with a regular end of treatment (EOT). (**B**) Swimmer plot showing the time on adjuvant anti-PD1 in weeks for patients that stopped treatment due to toxicity (n = 22). The vertical line indicates the median. (**C**) RFS for patients that stopped adjuvant anti-PD1 due to toxicity, stratified by the median time on treatment.

Patients who stopped adjuvant anti-PD1 treatment due to toxicity (n = 22) did not show a significantly worse RFS/OS compared to patients that completed adjuvant treatment (n = 119), neither in the uni- nor multivariate analysis (Figure 3A and Figure S1). However, there was a trend for relapses to occur earlier (1-year RFS rate 77.3% vs. 98.3%). Patients who stopped treatment due to toxicity had a median treatment duration of 12 weeks (range 1–55) (Figure 3B). In patients who received less than 12 weeks of treatment (n = 10), a significantly reduced RFS was observed (mRFS 21.6 months, *p* = 0.0023), compared to patients who received at least 12 weeks of treatment before stopping due to toxicity (n = 12) (Figure 3C). In fact, relapse or death events were only recorded in the former group, but none in the latter.

### 3.2. Relapsing Disease after Adjuvant Treatment

Follow-up data of 143 patients that suffered from a relapse in the adjuvant setting were available (Table 3). The majority were cutaneous melanomas (90.9%) and the relapses were most commonly preceded by adjuvant anti-PD1 (76.2%), anti-CTLA4 (11.8%), or BRAF/MEKi (7%). Relapses were distant metastases in most cases (51.0%) and occurred across a large timespan after the initiation of adjuvant treatment (median 6.2 months, range 0.3–47.2); 27.8% were within the first 3 months, 36.8% were between 3 and 12 months, and 35.4% were after 12 months. In 37.1% of cases, the relapsing melanoma was deemed resectable. A subset of patients with resectable disease went on to receive a second adjuvant treatment, such as BRAF/MEKi (n = 15, 28.3% of resectable cases) or anti-PD1 (n = 7, 13.2%) treatments; the other cases were followed-up with no systemic therapy (n = 31, 58.5%). Most relapses, however, were unresectable and these patients received a 1L metastatic treatment, such as anti-CTLA4/PD1 combination treatment (n = 25, 30.9%), BRAF/MEKi treatment (n = 20, 24.7%), or anti-PD1 treatment (n = 8, 9.9%), or they were enrolled in a clinical trial (n = 14, 17.3%).

**Figure 4:**
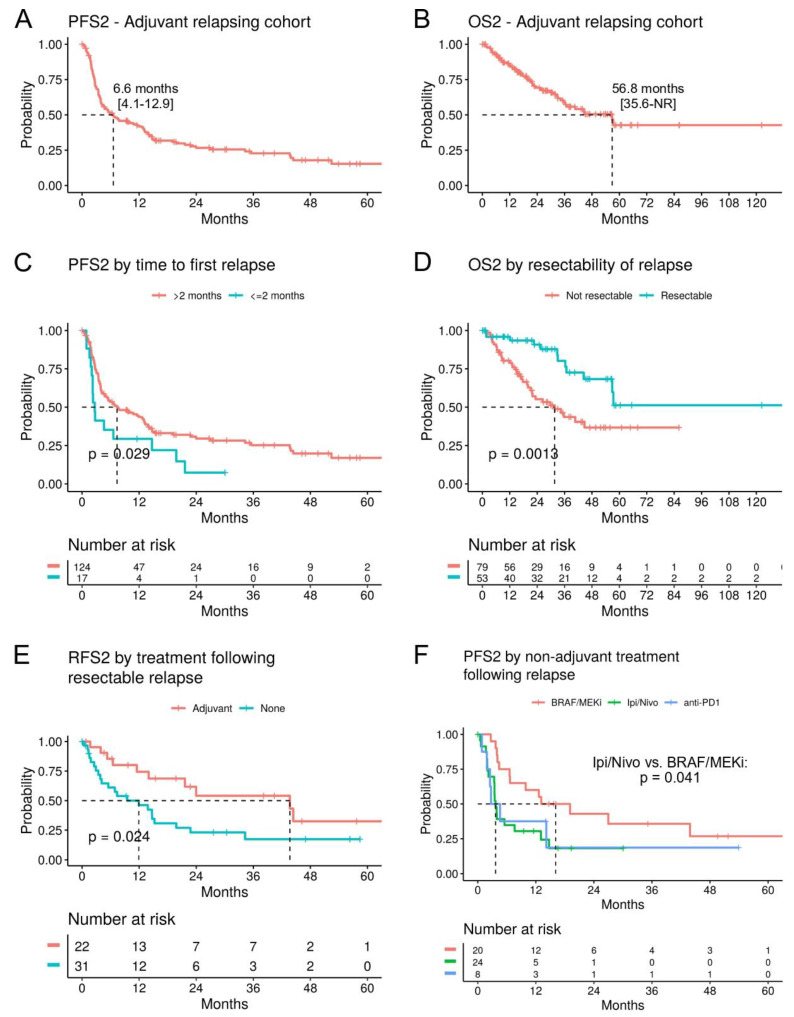
Survival analysis after relapse on adjuvant treatment. (**A**) Kaplan–Meier curves for the second progression-free survival (PFS2) and (**B**) OS2 following relapse during the first adjuvant treatment (n = 141). (**C**) PFS2 stratified by time until relapse during the first adjuvant treatment had occurred. (**D**) OS2 by resectability of the relapse during the first adjuvant treatment. (**E**) RFS2 for patients with a resectable relapse, stratified by a second adjuvant treatment versus follow-up only. (**F**) PFS2 for patients with a 1L metastatic treatment following the relapse, stratified by treatment.

Relapsing disease after adjuvant treatment is a challenging scenario, as demonstrated by the median progression-free survival 2 (mPFS2) of 6.6 months (95% CI; 4.1–12.9) and mOS2 of 56.8 months (95% CI; 35.6-NR) in patients with cutaneous or MUP (n = 141) (Figure 4A,B). Interestingly, in contrast to the first adjuvant treatment, the site of relapse did not correlate with survival. However, early relapses (within the first 2 months of adjuvant treatment, n = 17) proved particularly difficult, with a significant reduction in the second PFS (PFS2, HR 1.83, *p* = 0.029) (Figure 4C) and a numerical trend for reduced OS2 (HR 1.77, *p* = 0.12), compared to relapses that had occurred after 2 months (n = 124). As expected, patients with a resectable relapse (n = 53) had a significantly improved PFS2 (HR 0.53, *p* = 0.004) and OS2 (HR 0.34, *p* = 0.0013) (Figure 4D).

In patients with a resectable relapse, a second adjuvant treatment (n = 22) still showed efficacy with a mRSF2 of 43.7 months (95% CI; 21.6-NR), compared to 11.9 months (95% CI; 4.1–22.8) for follow-up only (n = 31; *p* = 0.024) (Figure 4E). The comparison of second adjuvant BRAF/MEKi versus anti-PD1 treatments did not show a significant difference.

For the analysis of 1L metastatic treatment following adjuvant relapse, the approved BRAF/MEKi, anti-CTLA4/PD1, and anti-PD1 drugs were included (n = 52). This population faces a poor prognosis, with a mPFS2 of 6.6 months (95% CI; 3.9–14.7) and a mOS2 of 34.3 months (95% CI; 23.3-NR) (Supplementary Figure S2). Treatment with anti-CTLA4/PD1 (n = 24) was associated with significantly worse PFS2 (*p* = 0.041) compared to BRAF/MEKi (n = 20) (Figure 4F), but this did not translate to an independent association with OS2 in a multivariate analysis including brain metastasis and the prior adjuvant therapy (Supplementary Figure S3). The strongest predictor of OS2 was elevated LDH levels (HR 9.6, *p* = 0.019) (Supplementary Figure S3).

### 3.3. Unresectable Setting: First-Line Metastatic Treatment

A total of 375 patients received a 1L treatment in the unresectable setting, of which 72.3% had a cutaneous melanoma, 12.0% had a MUP, 9.1% had a uveal melanoma, and 6.7% had a mucosal melanoma (Table 4). The majority of patients were stage 4 (82.1%). Distant metastases were most commonly located in the lungs (42.7% of patients), followed by liver (26.4%), brain (20.5%), and bone (20.3%) metastases. The most frequent treatments were ICIs (anti-PD1 monotherapy or anti-CTLA4/PD1 combination in 34.1% and 31.2%, respectively), followed by BRAF/MEKi (11.2%).

**Figure 5:**
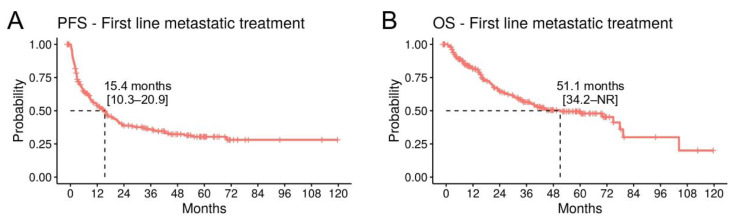
Survival analysis in the metastatic/unresectable cohort. (**A**) Kaplan–Meier curves for PFS and (**B**) OS for patients with a cutaneous or unknown primary melanoma treated with anti-PD1, anti-PD1/anti-CTLA4, or BRAF/MEKi in the metastatic setting (n = 235).

For the survival analyses, we included patients with a cutaneous or MUP treated with anti-PD1 (n = 113), anti-CTLA4/PD1 (n = 82), and BRAF/MEKi (n = 40). Overall (n = 235), the mPFS was 15.4 months (95% CI; 10.3–20.9) and the mOS was 51.1 months (95% CI; 34.2-NR) (Figure 5A,B). The 5-year OS rates were 46.5% (95% CI; 36.3–59.6) for anti-PD1, 49.2% (95% CI; 34.9–69.3) for BRAF/MEK inhibitors, and 52.4% (95% CI; 40.9–67.3) for anti-CTLA4/PD1 (Table 5). Treatment did not significantly correlate with survival, but there was a trend for improved OS on anti-CTLA4/PD1 in a multivariate analysis (HR 0.64, *p* = 0.15), compared to anti-PD1 (Supplementary Figure S4). Elevated LDH levels (HR 2.01, *p* = 0.013) and the presence of brain metastases (HR 1.78, *p* = 0.013) were significantly correlated with a worse OS (Supplementary Figure S4).

### 3.4. Unresectable Setting: Second-Line Metastatic Treatment

**Figure 6:**
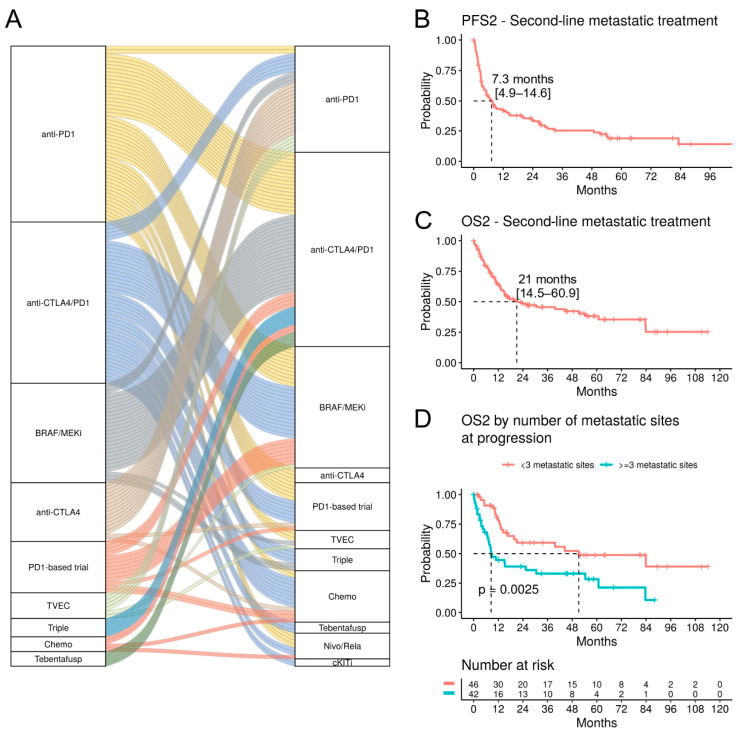
Survival analysis in the second-line metastatic treatment. (**A**) Alluvial plot showing the first and second-line treatment. Each band indicates one patient. (**B**) Kaplan–Meier curves for PFS2 and (**C**) OS2 for patients with a cutaneous or unknown primary melanoma treated with anti-PD1, anti-PD1/anti-CTLA4, or BRAF/MEKi in the second-line metastatic setting (n = 101). (**D**) OS2 stratified by number of metastatic sites at progression during the first-line metastatic treatment.

Follow-up data from 169 patients with progressive disease requiring a second-line (2L) treatment was available (Table 6). Of those, 72.8% had cutaneous melanoma and 10.1% had a MUP. The proportion of patients with elevated LDH levels nearly doubled compared to the 1L setting (from 18.4% to 34.3%) (Table 3 and Table 4). The most common 2L treatment was the anti-CTLA4/PD1 combination treatment (31.4%), and the majority of these patients had received 1L BRAF/MEKi (39.6%) or anti-PD1 inhibitors (32.1%) (Figure 6A). 2L BRAF/MEKi treatment (19.5%) was most frequently preceded by the failure of 1L anti-CTLA4/PD1 treatment (42.4%) or anti-PD1 monotherapy (33.3%). 2L anti-PD1 monotherapy (17.2%) was initiated most frequently after failure of anti-CTLA4 monotherapy (48.3%).

For the survival analyses, we included patients with a cutaneous melanoma or MUP treated with anti-PD1, anti-CTLA4/PD1, and BRAF/MEKi (n = 101). The therapeutic efficacy in the 2L setting is clearly reduced, as shown by the mPFS2 of 7.3 months (95% CI; 4.9–14.6) and mOS2 of 21 months (95% CI; 14.5–60.9) (Figure 6B,C). 2L anti-PD1 treatment (n = 29) was correlated with an improved OS2 compared to BRAF/MEKi or anti-CTLA4/PD1 treatments (mOS 83.9, 15.2, 12.5 months, respectively; *p* < 0.001, pairwise comparison) (Supplementary Figure S5); however, the majority of those patients had received a 1L anti-CTLA4 treatment, which has low benefits as a monotherapy. Indeed, in a multivariate analysis, the 1L anti-CTLA4 treatment was associated with a significantly improved OS2 (HR 0.09, *p* = 0.023), but 2L anti-PD1 treatment was not (Supplementary Figure S6).

A striking reduction in OS2 was observed in patients with at least three metastatic sites (n = 42) compared to patients with less widespread disease (mOS2 8.5 vs. 51.2 months, *p* = 0.0025) (Figure 6D). Particularly, bone metastases were associated with a worse OS2 (HR 2.62, *p* = 0.026) in a multivariate analysis (Supplementary Figure S6). Furthermore, OS2 was strongly correlated with elevated LDH levels (HR 4.65, *p* < 0.001) (Supplementary Figure S6).

## 4. Discussion

In this large single-center study, we report and analyze data on the real-world survival outcomes of patients with advanced melanoma, which have largely been improving over the last decade. In unresectable cases, treatment with ICIs and BRAF/MEKi resulted in a mOS of more than 4 years and a mPFS of 15.4 months. The 5-year PFS rates in the 1L treatment were 32.2% for anti-PD1, 30.2% for anti-CTLA4/PD1, and 29.7% for BRAF/MEKi; the 5-year OS rates were 46.5%, 52.4%, and 49.2%, respectively. These results are in line with the outcomes in the phase 3 trials, confirming the significant benefit that has been achieved in daily practice, despite the strict inclusion and exclusion criteria typically used in clinical trials [24,25].

Approximately two thirds of our patients received ICIs in the 1L metastatic/unresectable setting and only 11.2% patients started with BRAF/MEKi, corresponding to the currently established strategy with ICIs as the preferred 1L treatment option worldwide. In the multivariate analysis, high LDH levels and the presence of brain metastases were strongly correlated with a poorer OS. This is in line with multiple prospective trials suggesting elevated LDH levels as one of the most important prognostic and predictive biomarkers for impaired survival in metastatic melanoma patients [3,6,26].

Regarding 1L treatment, there was a numerical trend for longer survival with anti-CTLA4/PD1 compared to anti-PD1 treatment (mOS NR vs. 39.6 months). Similarly, the Checkmate 067 trial, which revolutionized the treatment landscape in metastatic melanoma, reported a doubling of the mOS in the anti-CTLA4/PD1 combination arm compared to the nivolumab arm at the 6.5-year trial update (72.1 vs. 36.9 months, respectively); however, this study was not powered to detect any differences between those two arms [27]. On the other hand, particularly due to the higher rates of immune-related adverse effects observed with the combination treatment, the therapeutic choice between anti-CTLA4/PD1 and anti-PD1 treatments should be grounded on the following prognostic factors: LDH levels, the presence of brain metastases, and comorbidities such as the presence of autoimmune diseases ([28] and the current version of Swiss melanoma guidelines, unpublished).

In the 2L setting, anti-PD1-treated patients had a significantly longer OS2 (mOS2 83.9 months) versus BRAF/MEKi or anti-CTLA4/PD1-treated patients (mOS2 15.2 and 12.5 months, respectively), yet none of the 2L treatments were superior in a multivariate analysis controlling for age, gender, LDH, metastatic sites, and 1L treatment. However, the low number of patients limits conclusions regarding 2L therapy. In general, there is no firmly established approach in BRAF wildtype patients, who are not responding to frontline therapy due to acquired resistance, apart from inclusion in clinical trials. For BRAF mutant patients, the appropriate choice after combination ICI treatment failure is a BRAF/MEKi treatment, based on the prospective DREAMseq trial [18].

Notably, the 1L anti-CTLA4-based treatment was associated with an improved OS2 in the multivariate analysis, in line with prospective trials [17,18]. One possible explanation is that anti-CTLA4 has a priming effect that leads to better immunological responses during the ensuing anti-PD1 therapy, similar to anti-CTLA4 and anti-PD1 combination therapy. Another explanation is that tumors that progressed during anti-CTLA4 treatment are not as aggressive as tumors that escape the more effective treatments, like anti-PD1 and BRAF/MEKi treatments; therefore, the likelihood of a response to a 2L treatment would be greater.

In the adjuvant cohort, the mRFS was 50 months overall. The 3-year RFS rates were 53% for anti-PD1 and 67.6% for BRAF/MEKi treatments. This is comparable to the 3-year RFS rates reported in the three landmark trials Checkmate 238 (58%; adjuvant nivolumab), Keynote-054 (63.7%; adjuvant pembrolizumab), and COMBI-AD (59%; adjuvant dabrafenib/trametinib) [10,13,29]. The slightly lower RFS rates in our anti-PD1 cohort could be explained by the difference in clinical management, given that all patients in these trials have received complete lymphadenectomy prior to commencing adjuvant treatment, while this is not the case in our cohort. In addition, PD1 blockade dominated the adjuvant melanoma landscape with only 22 patients having received BRAF/MEKi, meaning that our adjuvant cohorts are not fully balanced, reflecting current preferences in medical care.

The presence of lymph node plus in-transit metastases prior to adjuvant treatment was significantly correlated with the risk for death but not with the risk for relapse. These patients had a significantly worse OS, similar to patients having distant resectable metastases, raising the question of whether a treatment escalation with an anti-CTLA4/PD1 combination according to the IMMUNED protocol would be justifiable [30]. The prognostic importance of patients with non-nodal metastases was underlined in the new staging edition with in-transit patients representing a new substage category “c” [31]. Nevertheless, a sub-analysis from the Checkmate 238 trial showed a similar efficacy of nivolumab in patients with or without in-transit metastasis [32]; the Keynote-054 trial excluded patients with in-transit metastasis.

Approximately 10% of our patients ended adjuvant treatment due to toxicity, which was considerably lower than reported in clinical trials or other retrospective studies [13,33], yet similar to the Checkmate 238 trial [11]. Treatment cessation due to toxicity reduces drug exposure and it is unclear whether this translates to an increased risk of relapse. Moreover, there are no prospective trials investigating the optimal duration of adjuvant treatment. In our cohort, there was no negative impact on RFS or OS overall for patients who stopped treatment due to toxicity in the anti-PD1 arm. However, conclusions are limited by the small number of patients in this group. It was noteworthy that all relapses in this group occurred in patients who received adjuvant treatment for less than 3 months, implying a minimum time on the drug of at least 3 months for a relevant reduction in relapse risk in the adjuvant setting. Schumann et al. reported an improved RFS in patients treated with anti-PD1 who experienced relevant toxicity compared to those with no toxicity, which has also been observed in the advanced setting [34,35]. This study included both patients that stopped treatment due to toxicity and patients with toxicity that continued the treatment.

One-hundred and forty-one patients with a cutaneous melanoma or MUP experienced a relapse in the adjuvant setting, with 1/3 of relapses occurring off-treatment. Patients who experienced a relapse within the first 2 months after commencing adjuvant treatment had a significantly reduced PFS2. Early relapses after adjuvant treatment represent the most challenging patients in everyday oncology practice since these patients are excluded from all current 1L clinical trials. Furthermore, elevated LDH levels at the time of first relapse were strongly correlated with poor outcomes. In our small cohort (n = 52 patients) of patients receiving 1L treatment after adjuvant treatment failure, BRAF/MEKi achieved a better PFS2 in comparison to anti-CTLA4/PD1—though this was not significant in the multivariate analysis including patients with brain metastases. In another multicenter retrospective trial that investigated the management of relapses after anti-PD1, BRAF/MEKi and anti-CTLA4-based treatments (mono or combi) showed the most utility [36]. LDH levels, time of relapse (early versus late), as well as disease kinetics should guide further clinical management.

Of the 53 patients that experienced a fully resectable relapse under adjuvant treatment, 22 (41.5%) patients went on a second course of adjuvant treatment, and the majority of those received BRAF/MEKi. The second course of adjuvant treatment—independent of treatment type—was still efficacious (mRFS2 43.7 months), albeit less so than the first adjuvant treatment (mRFS 50 months). While both anti-PD1 and BRAF/MEKi provided benefits as second-line adjuvant treatments in our analysis, due to the retrospective setting and small sample size, our analysis does not offer a clear suggestion between the two. Recently, a group from MIA explored the efficacy and safety of a second-line adjuvant treatment with BRAF/MEKi (n = 55) [37]. They showed a prolonged mRFS2 of 33.4 months while mOS2 was not reached [37]. While the RFS2 appears shorter during the second course of adjuvant treatment compared to the 1L adjuvant trials, it can be still considered as an option, mainly within clinical trials in high-risk populations experiencing resectable relapses after adjuvant treatment.

Our study has a number of limitations, including the inherent biases in retrospective studies. Findings from a single center may be influenced by local practices and specific patient demographics and therefore not fully represent broader populations or settings. Additionally, some subgroup analyses are limited by small patient numbers, especially in the second course of adjuvant treatment, 1L metastatic following an adjuvant treatment, and 2L setting. On the other hand, this study represents a robust and homogenous cohort of advanced melanoma patients treated with BRAF/MEKi and ICIs beyond clinical trials.

## 5. Conclusions

This study provides real-world insights into the clinical outcomes and management of metastatic melanoma patients treated with ICIs and BRAF/MEKi in both the adjuvant and unresectable setting in a tertiary referral center in Switzerland. LDH levels remain an important prognostic factor in the 1L setting and in patients relapsing during adjuvant treatment. Early adjuvant relapse patients represent a challenging group; unfortunately, these patients are still excluded from all 1L trials. A second course of adjuvant treatment could be discussed in the context of some cases of high-risk fully resectable relapses, ideally within clinical protocols. In the unresectable setting, the approved 1L treatments are very effective in the real-world setting, with 5-year OS rates around 50%. However, outcomes remain poor for patients with brain metastases or progression during 1L treatment.

## Figures and Tables

**Table 1:** Patients in the adjuvant cohort.

	Total (n = 331)	Cutaneous Melanoma (n= 301)	Melanoma of Unknown Primary (n = 24)	Mucosal Melanoma (n = 6)
**Age**				
Median [Min, Max]	62.0 [15.0, 87.0]	61.0 [15.0, 87.0]	67.5 [17.0, 79.0]	69.0 [33.0, 79.0]
**Sex**				
f	130 (39.3%)	120 (39.9%)	6 (25.0%)	4 (66.7%)
m	201 (60.7%)	181 (60.1%)	18 (75.0%)	2 (33.3%)
**BRAF mutation**				
V600	141 (42.6%)	131 (43.5%)	9 (37.5%)	1 (16.7%)
non-V600	18 (5.4%)	17 (5.6%)	1 (4.2%)	0 (0%)
wildtype	131 (39.6%)	115 (38.2%)	13 (54.2%)	3 (50.0%)
unknown	41 (12.4%)	38 (12.6%)	1 (4.2%)	2 (33.3%)
**Stage**				
Stage 1b	2 (0.6%)	0 (0%)	0 (0%)	2 (33.3%)
Stage 2a	1 (0.3%)	1 (0.3%)	0 (0%)	0 (0%)
Stage 2b	6 (1.8%)	6 (2.0%)	0 (0%)	0 (0%)
Stage 2c	7 (2.1%)	6 (2.0%)	0 (0%)	1 (16.7%)
Stage 3a	28 (8.5%)	27 (9.0%)	0 (0%)	1 (16.7%)
Stage 3b	118 (35.6%)	110 (36.5%)	8 (33.3%)	0 (0%)
Stage 3c	138 (41.7%)	124 (41.2%)	13 (54.2%)	1 (16.7%)
Stage 3d	8 (2.4%)	8 (2.7%)	0 (0%)	0 (0%)
Stage 4	23 (6.9%)	19 (6.3%)	3 (12.5%)	1 (16.7%)
**Metastatic sites**				
distant	21 (6.3%)	18 (6.0%)	3 (12.5%)	0 (0%)
In transit	64 (19.3%)	61 (20.3%)	3 (12.5%)	0 (0%)
lymph node	210 (63.4%)	194 (64.5%)	14 (58.3%)	2 (33.3%)
lymph node & in transit	19 (5.7%)	15 (5.0%)	4 (16.7%)	0 (0%)
Missing	17 (5.1%)	13 (4.3%)	0 (0%)	4 (66.7%)
**Adjuvant treatment**				
anti-PD1	248 (74.9%)	229 (76.1%)	16 (66.7%)	3 (50.0%)
Clinical trial	28 (8.5%)	26 (8.6%)	2 (8.3%)	0 (0%)
BRAF/MEKi	24 (7.3%)	21 (7.0%)	3 (12.5%)	0 (0%)
anti-CTLA4	23 (6.9%)	20 (6.6%)	3 (12.5%)	0 (0%)
anti-CTLA4/PD1	8 (2.4%)	5 (1.7%)	0 (0%)	3 (50.0%)
**End of Treatment reason**				
regular	151 (45.6%)	137 (45.5%)	11 (45.8%)	3 (50.0%)
progression	79 (23.9%)	72 (23.9%)	6 (25.0%)	1 (16.7%)
toxicity	36 (10.9%)	30 (10.0%)	5 (20.8%)	1 (16.7%)
other	18 (5.4%)	17 (5.6%)	0 (0%)	1 (16.7%)
Missing	47 (14.2%)	45 (15.0%)	2 (8.3%)	0 (0%)

**Table 2:** Survival times on adjuvant treatment.

Adjuvant Treatment		
	Anti-PD1 (n = 234)	BRAF/MEKi (n = 23)
Median RFS (95% CI)	50 months (28.3-NR)	57 months (27.5-NR)
		HR 0.63 (0.30–1.29), *p* = 0.2
1-year RFS (95% CI)	67.3% (61.4–73.7)	95.5% (87.1–100)
3-year RFS (95% CI)	53.0% (46.4–60.6)	67.6% (48.8–93.8)
Median OS (95% CI)	NR (NR-NR)	57 (57-NR)
		HR 1.10 (0.43–2.79), *p* = 0.84
1-year OS (95% CI)	95.0% (92.2–98.0)	100% (100–100)
3-year OS (95% CI)	83.2% (77.7–89.0)	89.1% (75.8–100)

95% confidence intervals indicated in brackets. RFS: relapse-free survival, OS: overall survival, HR: hazard ratio, NR: not reached.

**Table 3:** Patients with a relapsing disease in the adjuvant setting.

	Total (n = 143)
**Age**	
Median [Min, Max]	60.0 [17.0, 88.0]
**Sex**	
f	53 (37.1%)
m	90 (62.9%)
**Type**	
Cutaneous	130 (90.9%)
Unknown primary	11 (7.7%)
Mucosal	2 (1.4%)
**BRAF mutation**	
V600	79 (55.2%)
non-V600	9 (6.3%)
wildtype	55 (38.5%)
unknown	0 (0%)
**Time until relapse**	
<3 months	39 (27.3%)
3–12 months	53 (37.1%)
>12 months	51 (35.7%)
**Resectable relapse**	
Unresectable	81 (56.6%)
Resectable	53 (37.1%)
Missing	9 (6.3%)
**Subsequent systemic treatment**	
Adjuvant	22 (15.4%)
Non-adjuvant	74 (51.7%)
None	47 (32.9%)
**Clinical stage at relapse**	
Stage 3b	15 (10.5%)
Stage 3c	50 (35.0%)
Stage 3d	3 (2.1%)
Stage 4	75 (52.4%)
**Site of relapse**	
distant	73 (51.0%)
In transit	33 (23.1%)
lymph node	26 (18.2%)
lymph node & in transit	11 (7.7%)
**Locoregional metastatic sites**	
Mean (SD)	1.16 (0.373)
Median [Min, Max]	1.00 [1.00, 2.00]
**Distant metastatic sites**	
Mean (SD)	1.64 (0.948)
Median [Min, Max]	1.00 [1.00, 5.00]
**LDH levels**	
normal	87 (60.8%)
elevated	6 (4.2%)
Missing	50 (35.0%)
**2nd adjuvant treatment**	
BRAF/MEKi	15 (10.5%)
anti-PD1	7 (4.9%)
**1L metastatic treatment**	
Ipi/Nivo	25 (17.5%)
BRAF/MEKi	20 (14.0%)
Clinical trial	14 (9.8%)
anti-PD1	8 (5.6%)
anti-CTLA4	4 (2.8%)
MEKi	1 (0.7%)
TVEC	1 (0.7%)

**Table 4:** Patients in the metastatic cohort.

	Total (n = 375)	Cutaneous Melanoma (n = 271)	Melanoma of Unknown Primary (n = 45)	Uveal Melanoma (n = 34)	Mucosal Melanoma (n = 25)
**Age**					
Median [Min, Max]	67.0 [27.0, 95.0]	69.0 [30.0, 95.0]	61.5 [27.0, 90.0]	62.0 [37.0, 80.0]	71.0 [40.0, 90.0]
**Sex**					
f	137 (36.5%)	101 (37.3%)	12 (26.7%)	10 (29.4%)	14 (56.0%)
m	238 (63.5%)	170 (62.7%)	33 (73.3%)	24 (70.6%)	11 (44.0%)
**BRAF mutation**					
V600	127 (33.9%)	112 (41.3%)	13 (28.9%)	0 (0%)	2 (8.0%)
non-V600	25 (6.7%)	19 (7.0%)	3 (6.7%)	1 (2.9%)	2 (8.0%)
wildtype	220 (58.7%)	137 (50.6%)	29 (64.4%)	33 (97.1%)	21 (84.0%)
unknown	3 (0.8%)	3 (1.1%)	0 (0%)	0 (0%)	0 (0%)
**Stage**					
Stage 2	1 (0.3%)	0 (0%)	0 (0%)	0 (0%)	1 (4.0%)
Stage 3	65 (17.3%)	54 (19.9%)	6 (13.3%)	0 (0%)	5 (20.0%)
Stage 4	308 (82.1%)	216 (79.7%)	39 (86.7%)	34 (100%)	19 (76.0%)
Missing	1 (0.3%)	1 (0.4%)	0 (0%)	0 (0%)	0 (0%)
**LDH levels**					
elevated	69 (18.4%)	49 (18.1%)	6 (13.3%)	11 (32.4%)	3 (12.0%)
normal	255 (68.0%)	186 (68.6%)	31 (68.9%)	19 (55.9%)	19 (76.0%)
Missing	51 (13.6%)	36 (13.3%)	8 (17.8%)	4 (11.8%)	3 (12.0%)
**Distant metastatic sites**					
Median [Min, Max]	2.00 [1.00, 14.0]	2.00 [1.00, 14.0]	2.00 [1.00, 12.0]	1.00 [1.00, 8.00]	2.00 [1.00, 4.00]
**Lung metastasis**					
no	215 (57.3%)	146 (53.9%)	27 (60.0%)	26 (76.5%)	16 (64.0%)
yes	160 (42.7%)	125 (46.1%)	18 (40.0%)	8 (23.5%)	9 (36.0%)
**Liver metastasis**					
no	276 (73.6%)	214 (79.0%)	40 (88.9%)	1 (2.9%)	21 (84.0%)
yes	99 (26.4%)	57 (21.0%)	5 (11.1%)	33 (97.1%)	4 (16.0%)
**Brain metastasis**					
no	298 (79.5%)	213 (78.6%)	29 (64.4%)	32 (94.1%)	24 (96.0%)
yes	77 (20.5%)	58 (21.4%)	16 (35.6%)	2 (5.9%)	1 (4.0%)
**Bone metastasis**					
no	299 (79.7%)	213 (78.6%)	37 (82.2%)	26 (76.5%)	23 (92.0%)
yes	76 (20.3%)	58 (21.4%)	8 (17.8%)	8 (23.5%)	2 (8.0%)
**Treatment**					
anti-PD1	128 (34.1%)	100 (36.9%)	17 (37.8%)	3 (8.8%)	8 (32.0%)
anti-CTLA4/PD1	117 (31.2%)	69 (25.5%)	15 (33.3%)	19 (55.9%)	14 (56.0%)
BRAF/MEKi	42 (11.2%)	34 (12.5%)	7 (15.6%)	0 (0%)	1 (4.0%)
PD1-based trial	33 (8.8%)	28 (10.3%)	5 (11.1%)	0 (0%)	0 (0%)
anti-CTLA4	20 (5.3%)	19 (7.0%)	0 (0%)	0 (0%)	1 (4.0%)
TVEC	11 (2.9%)	11 (4.1%)	0 (0%)	0 (0%)	0 (0%)
Tebentafusp	10 (2.7%)	0 (0%)	0 (0%)	10 (29.4%)	0 (0%)
Triple	9 (2.4%)	8 (3.0%)	1 (2.2%)	0 (0%)	0 (0%)
Chemo	5 (1.3%)	2 (0.7%)	0 (0%)	2 (5.9%)	1 (4.0%)

**Table 5:** Survival times on metastatic treatment.

Non-Adjuvant Treatment			
	Anti-PD1 (n = 113)	Anti-CTLA4/PD1 (n = 82)	BRAF/MEKi (n = 40)
Median PFS (95% CI)	11.8 months (8.7–21.0)	13.5 months (5.8–35.0)	18.3 months (15.8-NR)
		HR 0.97 (0.68–1.4), *p* = 0.88	HR 0.80 (0.51–1.27), *p* = 0.35
1-year PFS (95% CI)	49.6% (41.0–60.0)	52.6% (42.7–64.9)	66.9% (53.7–83.4)
3-year PFS (95% CI)	34.1% (25.9–44.9)	36.6% (27.1–49.5)	40.1% (26.8–60.1)
5-year PFS (95% CI)	32.2% (23.9–43.4)	30.2% (20.7–43.9%)	29.7% (17.5–50.5)
Median OS (95% CI)	39.6 months (27.5-NR)	NR (38.1-NR)	42.4 (20.9-NR)
		HR 0.76 (0.48–1.20), *p* = 0.24	HR 0.93 (0.55–1.59), *p* = 0.8
1-year OS (95% CI)	81.8% (74.7–89.6)	84.8% (77.2–93.1)	73.9% (61.289.3)
3-year OS (95% CI)	52.1% (42.2–64.4)	61.9% (51.4–74.5)	55.8% (41.5–74.9)
5-year OS (95% CI)	46.5% (36.3–59.6)	52.4% (40.9–67.3)	49.2% (34.9–69.3)

95% confidence intervals indicated in brackets. PFS: progression-free survival, OS: overall survival, HR: hazard ratio, NR: not reached.

**Table 6:** Patients with a second-line metastatic treatment.

	Total (n = 169)	Cutaneous Melanoma (n = 123)	Uveal Melanoma (n = 18)	Melanoma of Unknown Primary (n = 17)	Mucosal Melanoma (n = 11)
**Age**					
Median [Min, Max]	65.0 [30.0, 91.0]	65.0 [31.0, 91.0]	61.0 [40.0, 74.0]	59.0 [30.0, 88.0]	69.0 [43.0, 77.0]
**Sex**					
f	61 (36.1%)	46 (37.4%)	7 (38.9%)	3 (17.6%)	5 (45.5%)
m	108 (63.9%)	77 (62.6%)	11 (61.1%)	14 (82.4%)	6 (54.5%)
**BRAF mutation**					
V600	71 (42.0%)	63 (51.2%)	0 (0%)	6 (35.3%)	2 (18.2%)
non-V600	13 (7.7%)	9 (7.3%)	1 (5.6%)	1 (5.9%)	2 (18.2%)
wildtype	85 (50.3%)	51 (41.5%)	17 (94.4%)	10 (58.8%)	7 (63.6%)
unknown	0 (0%)	0 (0%)	0 (0%)	0 (0%)	0 (0%)
**Stage**					
Stage 3	19 (11.2%)	16 (13.0%)	0 (0%)	3 (17.6%)	0 (0%)
Stage 4	150 (88.8%)	107 (87.0%)	18 (100%)	14 (82.4%)	11 (100%)
**LDH levels**					
elevated	58 (34.3%)	41 (33.3%)	9 (50.0%)	5 (29.4%)	3 (27.3%)
normal	97 (57.4%)	75 (61.0%)	8 (44.4%)	8 (47.1%)	6 (54.5%)
Missing	14 (8.3%)	7 (5.7%)	1 (5.6%)	4 (23.5%)	2 (18.2%)
**Distant metastatic sites**					
Median [Min, Max]	2.00 [1.00, 12.0]	2.50 [1.00, 12.0]	2.00 [1.00, 7.00]	2.00 [1.00, 12.0]	3.00 [1.00, 5.00]
**Lung metastasis**					
no	94 (55.6%)	64 (52.0%)	13 (72.2%)	12 (70.6%)	5 (45.5%)
yes	75 (44.4%)	59 (48.0%)	5 (27.8%)	5 (29.4%)	6 (54.5%)
**Liver metastasis**					
no	109 (64.5%)	90 (73.2%)	0 (0%)	14 (82.4%)	5 (45.5%)
yes	60 (35.5%)	33 (26.8%)	18 (100%)	3 (17.6%)	6 (54.5%)
**Bone metastasis**					
no	121 (71.6%)	88 (71.5%)	14 (77.8%)	13 (76.5%)	6 (54.5%)
yes	48 (28.4%)	35 (28.5%)	4 (22.2%)	4 (23.5%)	5 (45.5%)
**Brain metastasis**					
no	125 (74.0%)	82 (66.7%)	18 (100%)	15 (88.2%)	10 (90.9%)
yes	44 (26.0%)	41 (33.3%)	0 (0%)	2 (11.8%)	1 (9.1%)
**2L treatment**					
anti-CTLA4/PD1	53 (31.4%)	33 (26.8%)	7 (38.9%)	7 (41.2%)	6 (54.5%)
BRAF/MEKi	33 (19.5%)	31 (25.2%)	0 (0%)	2 (11.8%)	0 (0%)
anti-PD1	29 (17.2%)	28 (22.8%)	0 (0%)	1 (5.9%)	0 (0%)
Chemo	14 (8.3%)	2 (1.6%)	7 (38.9%)	2 (11.8%)	3 (27.3%)
PD1-based trial	13 (7.7%)	10 (8.1%)	1 (5.6%)	2 (11.8%)	0 (0%)
Nivo/Rela	7 (4.1%)	5 (4.1%)	0 (0%)	1 (5.9%)	1 (9.1%)
Triple	6 (3.6%)	6 (4.9%)	0 (0%)	0 (0%)	0 (0%)
TVEC	5 (3.0%)	4 (3.3%)	0 (0%)	1 (5.9%)	0 (0%)
anti-CTLA4	4 (2.4%)	3 (2.4%)	0 (0%)	1 (5.9%)	0 (0%)
Tebentafusp	3 (1.8%)	0 (0%)	3 (16.7%)	0 (0%)	0 (0%)
cKITi	2 (1.2%)	1 (0.8%)	0 (0%)	0 (0%)	1 (9.1%)

## Data Availability

The data presented in this study are available on request from the corresponding author.

## Supplementary Materials

The following supporting information can be downloaded at: https://www.mdpi.com/article/10.3390/cancers16050854/s1; Figure S1: Multivariate analysis of OS in the adjuvant cohort; Figure S2: Survival analysis in the first-line metastatic treatment following a relapse on adjuvant treatment. A) Kaplan–Meier curves for PFS2 and B) OS2 for patients with a cutaneous or unknown primary melanoma treated with anti-PD1, anti-PD1/anti-CTLA4m, or BRAF/MEKi after a relapse on adjuvant treatment (n = 52). (95% confidence intervals are indicated in square brackets. 1L: first line. NR: not reached.); Figure S3: Multivariate analysis of OS2 in the first-line metastatic treatment following a relapse on adjuvant treatment; Figure S4: Multivariate analysis of OS in the metastatic/unresectable cohort; Figure S5: Survival analysis in the second-line metastatic treatment. Kaplan–Meier curves for OS2 for patients with a cutaneous or unknown primary melanoma treated with second-line anti-PD1, anti-PD1/anti-CTLA4, or BRAF/MEKi after progression during first-line treatment. (2L: Second line. NS: not significant.); Figure S6: Multivariate analysis of OS2 in the second-line metastatic treatment.

## Abbreviations

1L	First line
2L	Second line
BRAF/MEKi	BRAF/MEK inhibitor
CI	Confidence interval
CTLA4	Cytotoxic T-lymphocyte-associated protein 4
HR	Hazard ratio
ICI	Immune checkpoint inhibitor
LAG3	Lymphocyte activation gene 3
MUP	Melanoma of unknown primary
NR	Not reached
OS	Overall survival
PD1	Programmed cell death protein 1
PFS	Progression-free survival
RFS	Relapse-free survival
TT	Targeted therapy
