# Supplementary material for: Real-World Data on Clinical Outcomes and Treatment Management of Advanced Melanoma Patients: Single-Center Study of a Tertiary Cancer Center in Switzerland
Source: Cancers (Basel). 2024 Feb 20;16(5):854. doi: 10.3390/cancers16050854 (PMC10930974; doi:10.3390/cancers16050854)
Supplement: Supplementary file 1 [file cancers-16-00854-s001.zip › cancers-2876217 Supplementary figures.docx]

Supplementary figures


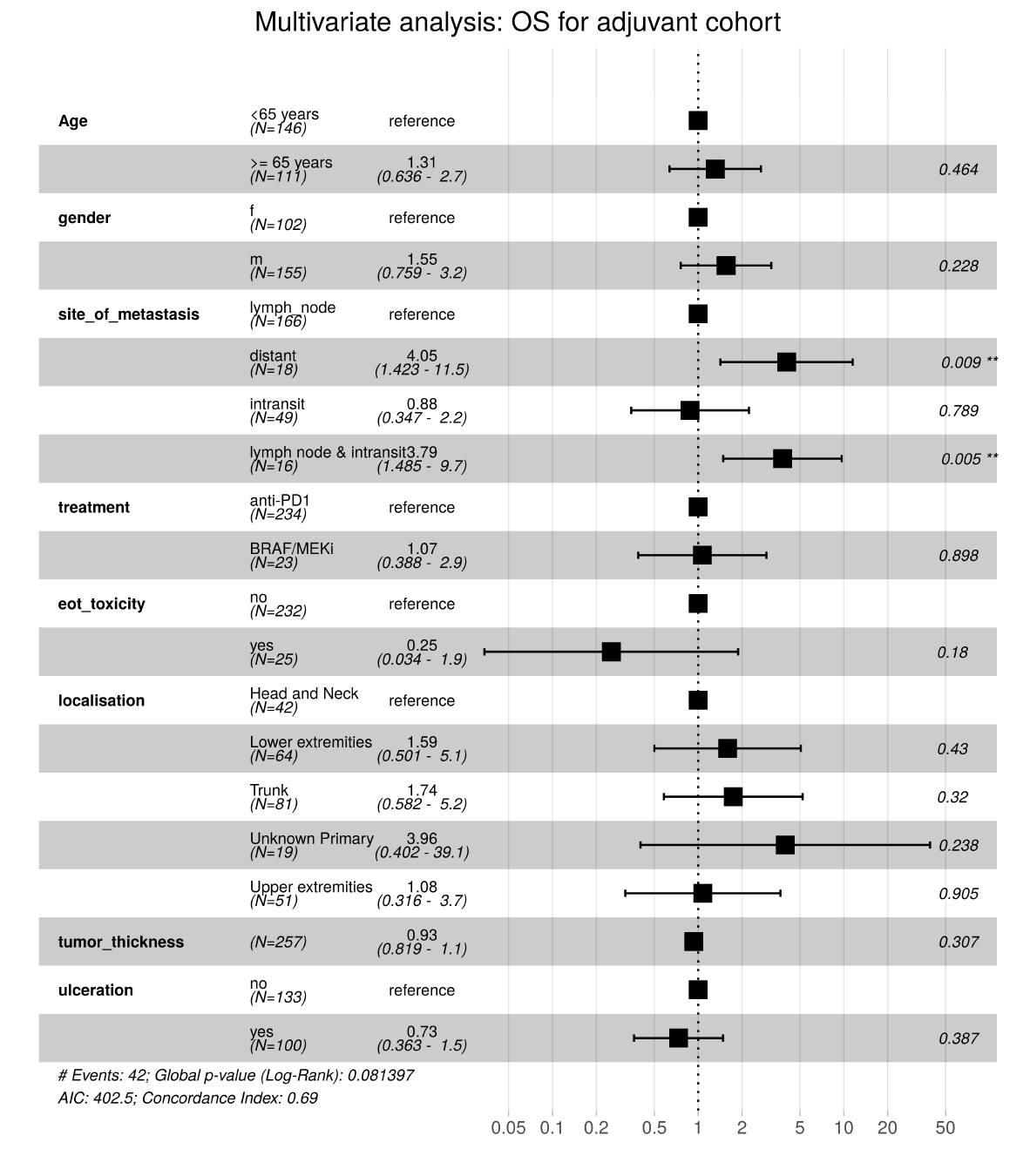


**Figure S1.** Multivariate analysis of OS in the adjuvant cohort. **: p≤0.005.


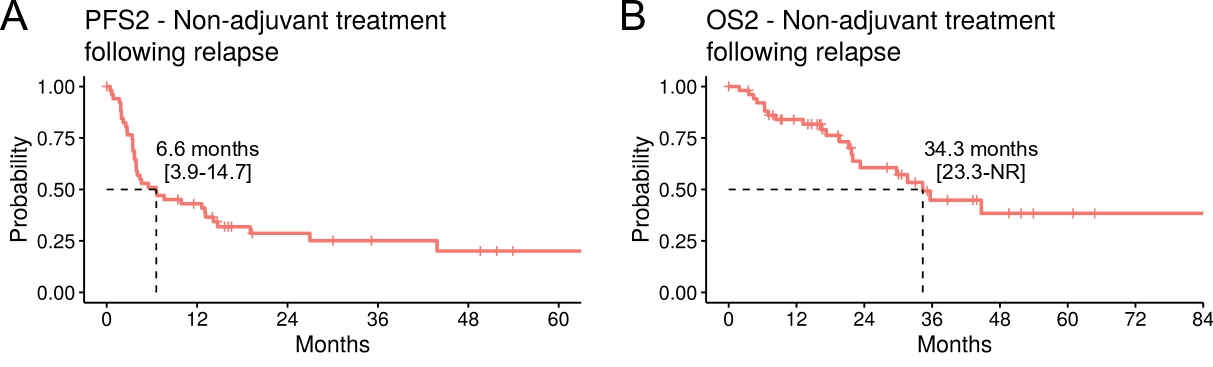


**Figure S2.** Survival analysis in the first-line metastatic treatment following a relapse on adjuvant treatment. A) Kaplan–Meier curves for PFS2 and B) OS2 for patients with a cutaneous or unknown primary melanoma treated with anti-PD1, anti-PD1/anti-CTLA4m, or BRAF/MEKi after a relapse on adjuvant treatment (n = 52). (95% confidence intervals are indicated in square brackets. 1L: first line. NR: not reached.).


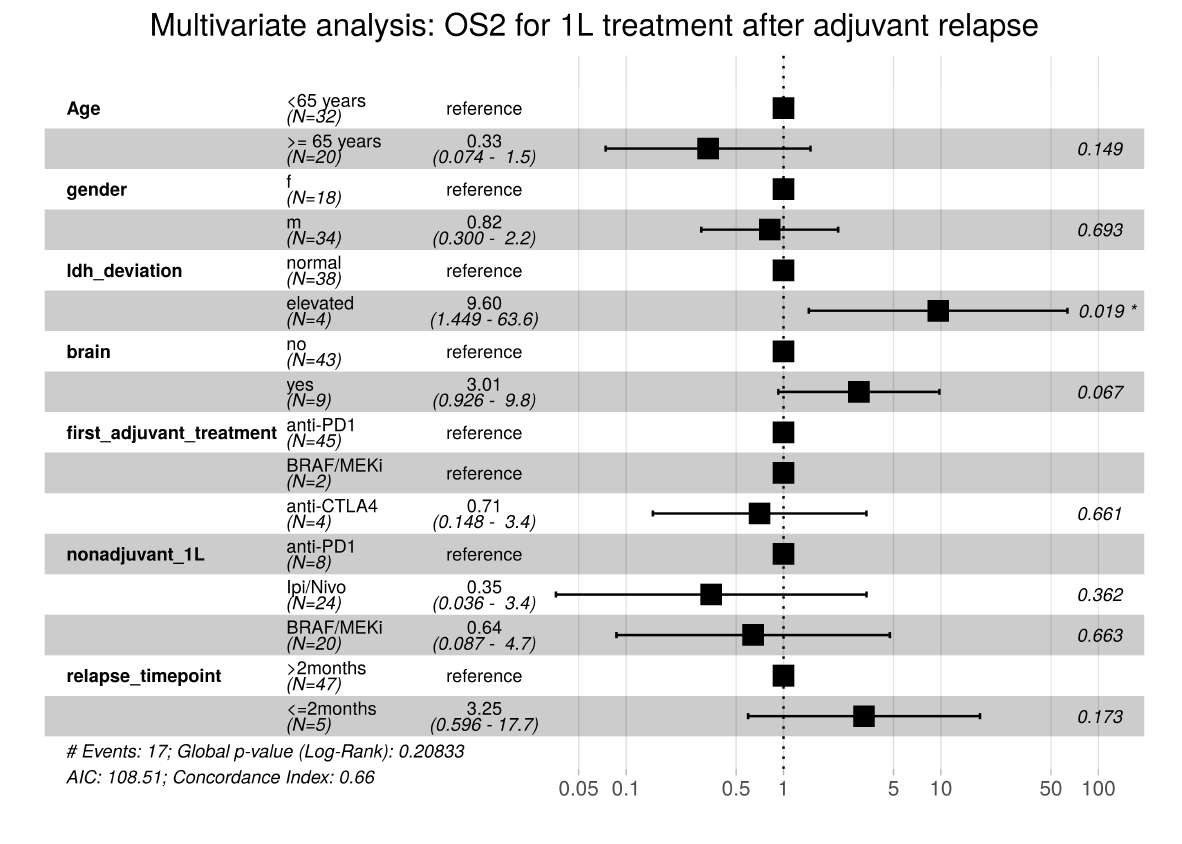


**Figure S3.** Multivariate analysis of OS2 in the first-line metastatic treatment following a relapse on adjuvant treatment. *: p≤0.05.


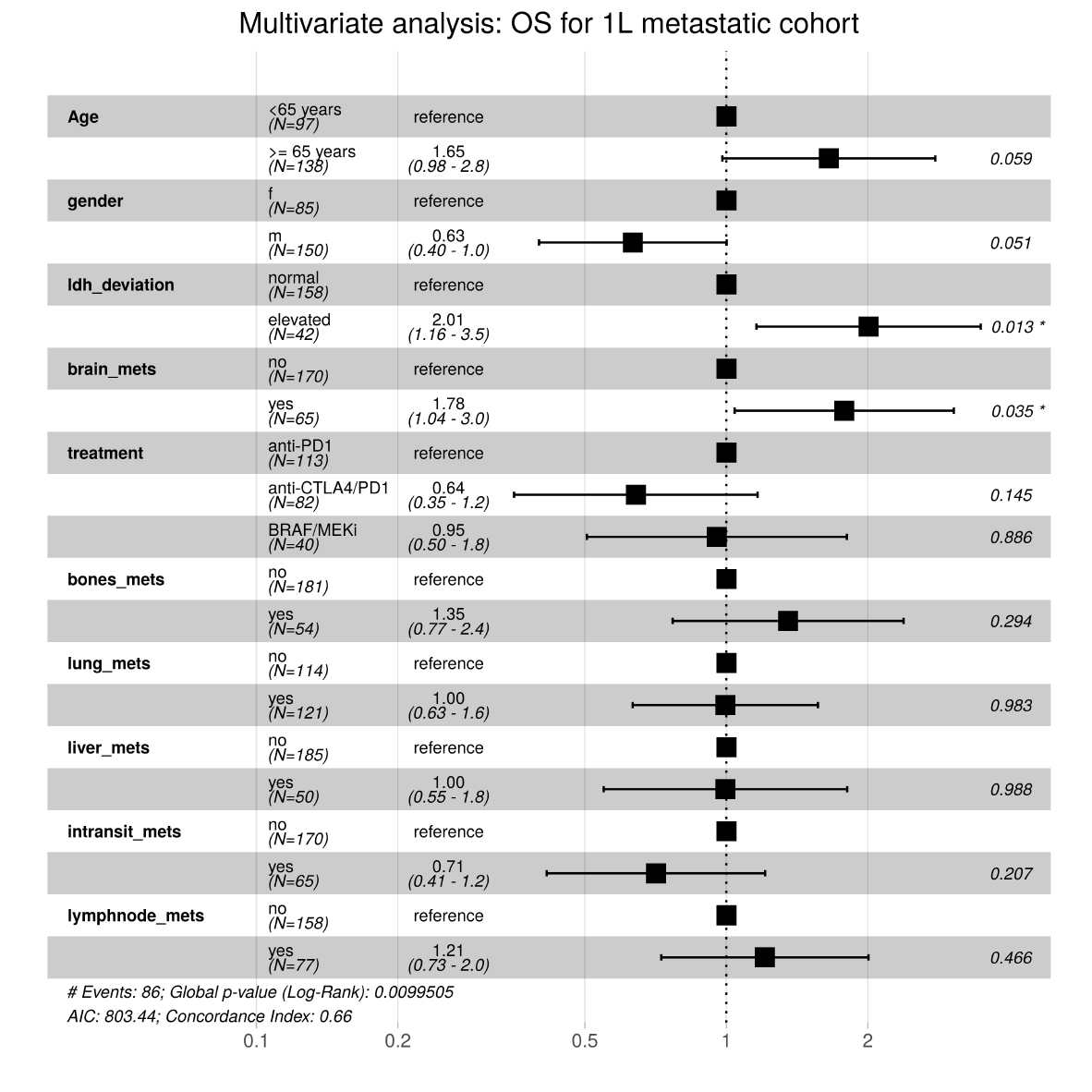


**Figure S4.** Multivariate analysis of OS in the metastatic/unresectable cohort. *: p≤0.05.


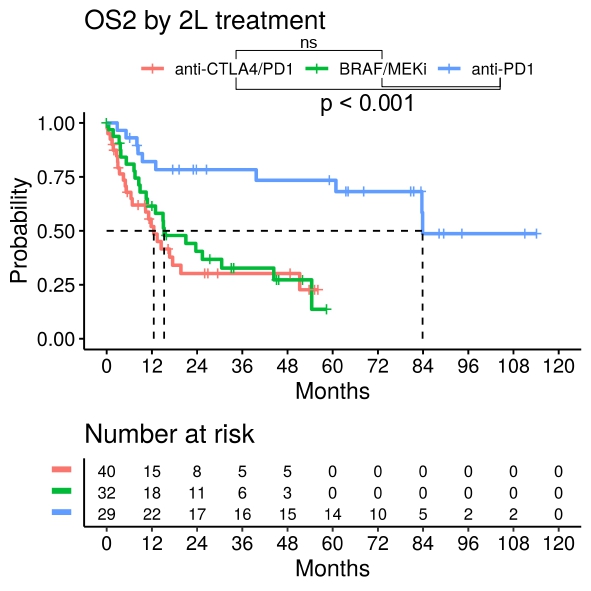


**Figure S5.** Survival analysis in the second-line metastatic treatment. Kaplan–Meier curves for OS2 for patients with a cutaneous or unknown primary melanoma treated with second-line anti-PD1, anti-PD1/anti-CTLA4, or BRAF/MEKi after progression during first-line treatment. (2L: Second line. NS: not significant.).


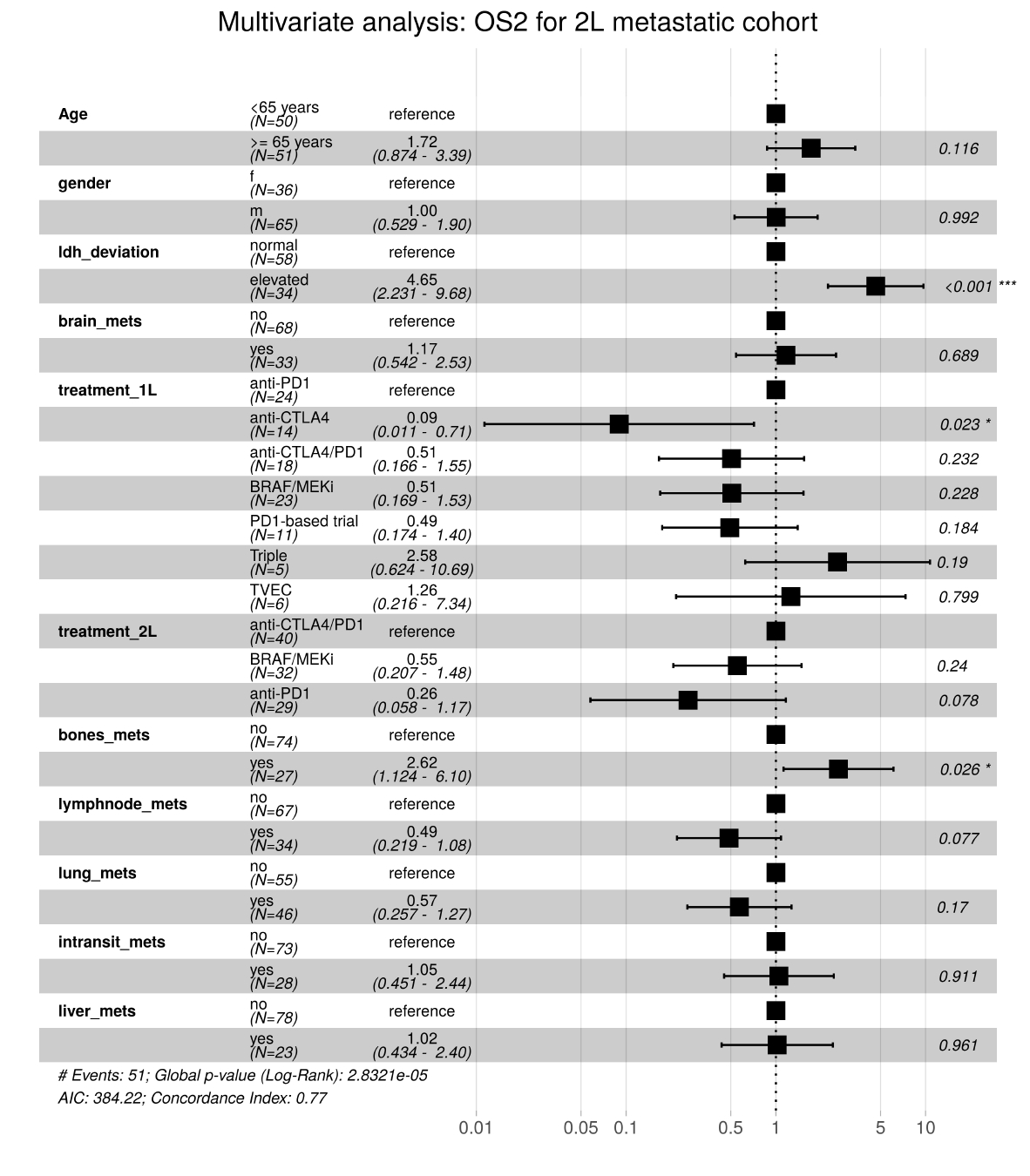


**Figure S6.** Multivariate analysis of OS2 in the second-line metastatic treatment. *: p≤0.05, ***: p≤0.001.
